# Migraine, tension-type headache, and depression among Saudi female students in Taif University

**DOI:** 10.1186/s42506-019-0008-7

**Published:** 2019-01-29

**Authors:** Dalia E. Desouky, Hany A. Zaid, Azza A. Taha

**Affiliations:** 1 Department of Family and Community Medicine, College of Medicine, Taif University, Alsalama street, Taif city, Saudi Arabia; 2 Department of Public Health and Community Medicine, Faculty of Medicine, Menoufia University, Shibin El Kom, Egypt

**Keywords:** Migraine, Headache, Depression, Saudi, Female, University

## Abstract

**Background:**

Studies done in Saudi Arabia showed a high prevalence of headache among university students. Limited research was done to assess the relationship between headache and psychiatric disorders. The aim of this study was to assess the prevalence and association between migraine, tension-type headache, and depression among Saudi female students in Taif University.

**Participants and methods:**

A cross-sectional study using self-administered questionnaires about headache and depression was conducted at the Taif University on 1340 female students in the academic year 2016–2017. The Beck Depression Inventory, the ID Migraine™ screening tool, and the criteria of the International Headache Society were used to investigate the depressive symptoms and headache types.

**Results:**

The self-reported headache prevalence was 68.4%, and the prevalence of migraine, tension-type headache (TTH), and depression was 32.5%, 29.5%, and 6.2%, respectively. The main migraine trigger was stress or anxiety; 86.6% of migraineurs had a positive family history, and only 11.9% sought medical care for headache. Of students with TTH, 61.1% reported family history and only 12.4% sought medical care. Paracetamol was the commonly used analgesic for all headache types. Medical students and students in older grades showed significantly higher levels of all headache types. Depression prevalence was significantly higher among migraineurs and students who suffered higher headache frequencies.

**Conclusion:**

The study demonstrated a high prevalence of headache among the studied students and an association between headache and depression. The study calls for increasing awareness towards headache and the importance of seeking medical consultation. Management strategies should be planned for the observed headache and depression comorbidity.

## Introduction

Primary headache disorders including migraine and tension-type headache (TTH) are of great importance to global public health due to its high prevalence [1]. Migraine which is the main cause of headache worldwide is characterized by headache attacks that last between 4 and 72 h if untreated. It is one-sided pulsating or throbbing pain of moderate to severe intensity and associated with nausea or vomiting or both photophobia and phonophobia [2]. Tension-type headache (TTH) is a headache where the pain is commonly described as “a band around the head”. It has at least two of the following characteristics: mild to moderate in intensity, occurs on both sides of the head (bilateral), and not worsened by routine activity (bending over or climbing stairs), and pain has a pressing or tightening quality and not throbbing or pulsing. It is also not accompanied by nausea or vomiting [3].

Previous studies have shown a high prevalence of headache among university students [4–8]. Students’ lifestyle puts them at high risk to suffer from fatigue, stress, and anxiety which are the most common causes of TTH and migraine [9]. Headache can make students suffer lost days of study, impaired academic performance, and poor quality of life [9]. The prevalence of migraine and TTH was reported to be higher among women [4, 10]. This was explained by the effect of female hormone levels, particularly estrogen [11].

Migraine and TTH sufferers were found to have a higher risk to develop depression [12, 13]. Females suffer a higher prevalence of depression more than men, and they have an obvious disability due to harder pain attacks. That is why women suffer more headache and depression comorbidity [14]. On the other hand, psychiatric disorders as depression are associated with increased headache-related disability, frequency, severity and risk for chronicity. In addition, these disorders lower the overall quality of life among migraine and TTH sufferers [15].

In the Kingdom of Saudi Arabia (KSA), a country-wide population-based cross-sectional survey was done on 2421 adult population (18–65 years) in 2013 and found a 32% of 1 year prevalence of migraine headache [16]. Regarding the university students, a study on 400 female university students of King Saud University (2008) reported a prevalence of 25.7% of migraine [17]. A study done in King Faisal University (2013) on 100 female students showed a prevalence of 58.4% and 41.6% of migraine and TTH, respectively [7]. Another study was conducted on female medical students at Taibah University (2015) and found a history of headache in 92% of students and TTH prevalence of 58% [18]. A recent study among female students at Taibah University (2016) demonstrated a prevalence of 61.8% of migraine among the participants [19].

A literature search of published studies showed that no study was done to assess the comorbidity of migraine, TTH, and depression among Saudi female university students. The aim of this study was to assess this comorbidity among female students at Taif University and the associated factors.

## Participants and methods

### Study design and time frame

A cross-sectional study was carried out on female students at Taif University in the period from February to June 2017.

### Sampling

Multistage sampling methodology was carried out where Taif University was chosen. The university community of the female section of Taif University was the sampling frame. The university includes four medical colleges and seven non-medical colleges for females. The target population included all female students of the eleven colleges.

The total number of female students registered in the eleven colleges in the academic year 2016–2017 was 1733. A total coverage was done, and all students in the 11 colleges in all grades and classes were contacted. For each college, the Office of Student Affairs was contacted and a student list was obtained for every grade. With the help of the coordinator and the leader of every grade, questionnaire copies equal to the students’ number in every grade were distributed by the researchers in the self-study session of students where they were free from any academic duty. After excluding non-respondents, and those who met the exclusion criteria (those with a past history of head or neck injury, nasal allergy or inflammation, headache of secondary neurological disorders, or systematic disease and pregnant students), the response rate was 77.3%, and the total number of participants in the study was 1340 students.

The number of the eligible participants from each faculty was as follows: Faculty of Medicine (198 students), Faculty of Pharmacy (183), Faculty of Applied Medical Sciences (180), Faculty of Dentistry (157), Faculty of Science (85), Faculty of Literature (80), Faculty of Sharia and Law (96), Faculty of Education (86), Faculty of Design and Home Economics (94), Faculty of Computing and Information Technology (93), and Faculty of Managerial And Financial Sciences (88).

### Study instrument

The study instrument was a pre-designed self-administered questionnaire that gathered information about age, college type, marital status, and educational grade. A stepwise approach was carried out to assess the prevalence of different types of headache, where a questionnaire to screen “headache” that contained three sections was used. The first section included a question to screen headache students, the second section included three questions to screen migraineurs, and the third section included four questions to screen students with TTH.

Step 1: For the determination of students with headache, an initial screening was carried out, where students who replied “yes” to this question: “Did you have two or more headaches in the last 3 months?” were the subjects with headache under consideration (916 students).

Step 2: For the screening of students who have migraine, students of positive answers in step 1 (916 students) were asked the three-item screening questions of the identification of migraine (ID Migraine™ screening tool): During the last 3 months, (1) Did you feel nauseated or sick in your stomach with your headaches? (2) Did light bother you when you had a headache (a lot more than when you do not have headaches)? and (3) Did your headache limit your ability to work, study, or do what you needed to do for at least 1 day? A test-diagnosis of migraine headache required at least two positive responses (436 students) [20].

The ID Migraine™ is a widely used screening tool for migraine that has showed good validity for diagnosis of migraineurs at primary health care services, as it investigates the major aspects of migraine headache, which are nausea, photophobia, and disability [20]. This tool was developed and validated by Lipton et al. and can be applied quickly to large numbers of populations [21]. Sensitivity, specificity, and positive predictive value of this test in primary care were estimated to be 81%, 75%, and 93%, respectively [22]. This test was also validated to be applied to adolescent students with a sensitivity of 62.1% and specificity of 71.1% [20–22]. In addition, it was used in studies done on university students in Arab countries and in Saudi Arabia [10, 19, 23].

For measuring migraine severity, pain intensity was measured on a 4-point scale ranged from 0 to 3, where 0 = no pain, 1 =mild, 2 = moderate, and 3 = severe headache. This scale was recommended by the International Headache Society [22]. For assessing migraine triggers, a list of triggers was provided in the questionnaire and included stress or anxiety, irregular sleep, much reading, exams, smoking, menstruation, exposure to sun, and noise. The students were instructed about the possibility to give more than one answer.

Step 3: For the diagnosis of TTH, the criteria of the International Headache Society (IHS) was used, where students screened positive in step 1, and did not fulfill the headache criteria of step 2, were asked if headache is (1) of pressing (non-pulsating) quality, (2) of mild or moderate intensity (may inhibit but does not prohibit activities), (3) of bilateral location, and (4) not aggravated by climbing stairs or similar routine physical activity. Diagnosis of TTH required at least two positive answers to the previous four questions (396 students) [24].

In the second and third sections, questions on frequency of headache, family history, medical consultation for headache, medications used, frequency of analgesic use, increase in headache frequency after analgesic use, and increasing analgesic dose used over time were presented. For students who reported having headache but who did not fulfill any of the migraine or TTH criteria, they were classified as students with unknown headache (84 students).

To assess the prevalence of depression among students, Beck Depression Inventory (BDI) was used. It is a 21-item scale where each item was scored from 0 to 3 according to the symptom severity, with a total score ranging from 0 to 63. It was possible to give an answer with a score that ranged from 0 to 3 (absent, mild, moderate, and severe). The patient was diagnosed as normal if having a score less than 26, mild depression if the score ranged from 26 to 38, moderate if ranged from 39 to 55, and severe depression if ranged from 56 to 63 [25].

### Statistical analysis

The data were coded, tabulated, and analyzed using the Statistical Package for the Social Sciences (SPSS, version 20; IBM Corp., Armonk, NY, USA). Qualitative data were expressed as numbers and percentages, and the chi-square (*χ*^2^) test was used to test the relationship between variables. Quantitative data were expressed as mean and standard deviation (mean ± SD). A *p* value of < 0.05 was considered as statistically significant.

## Results

Table 1 shows that the total number of the participants was 1340 female students, their mean age was 21.17 ± 2.2 years, 53.6% of them were from medical colleges and 89.9% were unmarried. Of the studied students, 68.4% reported having headache 2-3 times in the last 3 months, of them, 47.6% had migraine headache, 43.2% had TTH and 9.2% had unknown headache. No one was classified to have both migraine and TTH. Depression prevalence among studied students was 38.7%.

**Table 1:** Distribution of the studied female university students according to their personal characters, headache, and depression prevalence, Taif University, KSA, 2017

Variable	Female university students (*n* = 1340)	
	No.	%
College		
- Medical	718	53.6
- Non-medical	622	46.4
Age (mean ± SD)	21.17 ± 2.2 (range = 22–26)	
Marital status		
- Married	136	10.1
- Not married	1204	89.9
Grade		
- 1st grade	286	21.3
- 2nd grade	178	13.3
- 3rd grade	220	16.4
- 4th grade	178	13.3
- 5th grade	222	16.6
- 6th grade	256	19.1
Smoking		
- Yes	112	8.4
- No	1228	91.6
Having headache 2–3 times in the last 3 months		
- No	424	13.6
- Yes^±^	916	68.4
○Migraine	436	32.5
○TTH	396	29.5
○Unknown headache	84	6.2
Depressive symptoms		
- Present	519	38.7
- Not present	821	61.3

^±^Percentages were calculated from the total sample

In Table 2, 85.1% of migraineur students stated that headache limited their ability to study or enjoy life, and 94.7% reported that their headache was of moderate to severe intensity. The main trigger for migraine attacks was stress or anxiety, and 18.3% of migraineurs reported the presence of more than one trigger. Most of the migraineurs (43.6%) reported that the frequency of migraine attacks per month was fewer than daily to weekly, and 86.6% reported a family history of migraine.

**Table 2:** Distribution of migraineurs according to migraine characteristics, Taif University, KSA, 2017

Variable	Migraineur students (*n* = 436)	
	No.	%
Headache limit the ability to study or enjoy life		
- Yes	371	85.1
- No	65	14.9
Severity of migraine attacks		
- Mild	23	5.3
- Moderate	218	50.0
- Sever	195	44.7
Migraine triggers^#^		
- Stress or anxiety	225	51.6
- More than one answer	80	18.3
- Irregular sleep	48	11.0
- Menstruation	35	8.0
- Smoking	18	4.1
- Much reading	16	3.7
- Noise	10	2.3
- Exams	2	0.5
- Exposure to sun	2	0.5
Frequency of migraine per month^#^		
- Daily	53	12.2
- Fewer than daily to weekly	190	43.6
- Fewer than weekly to monthly	169	38.8
- Fewer than monthly to 1 year	24	5.5
Family history of migraine		
- Yes	200	45.9
- No	236	54.1
Seeking medical care for migraine		
- Yes	52	11.9
- No	384	88.1
Advice to taker analgesic was by^#^		
- Physician	61	14
- Pharmacist	33	7.6
- Family members	300	68.8
- Others: friends, colleagues, neighbors	42	9.6
Frequency of analgesic use		
- Daily	18	4.1
- Fewer than daily to weekly	409	93.8
- Fewer than weekly to monthly	7	1.6
- Fewer than monthly to 1 year	2	0.5
Increase in headache frequency after analgesic use		
- Yes	38	8.7
- No	398	91.3
Increase analgesic dose used over time		
- Yes	318	72.9
- No	118	27.1
Type of analgesic used^#^		
- Paracetamol	235	53.9
- Ibuprofen	141	32.3
- Acetaminophen	52	11.9
- Aspirin	6	1.4
- Diclofenac sodium	2	0.5

^#^Multiple responses

Only 11.9% of the migraineurs sought medical care, 68.8% took analgesic according to an advice from a family member, and only 21.6% of them took analgesic according to an advice from a physician or a pharmacist. In the vast majority of migraineurs (93.8%), the frequency of analgesic use was fewer than daily to weekly, with 72.9% of them reported an increase in the analgesic dose used over time. The main analgesic used was paracetamol (53.9%), followed by ibuprofen, acetaminophen, aspirin, and diclofenac sodium (32.3%, 11.9%, 1.4%, and 0.5%, respectively).

Table 3 shows that only 4.5% of students with TTH reported that headache affected their daily activities, 61.1% reported a family history of TTH, and the majority (71.7%) stated that the frequency of headache attacks per month was fewer than weekly to monthly. Only 12.4% of TTH students sought medical care, and 69.4% took analgesic according to an advice from a family member. The frequency of analgesic use was fewer than weekly to monthly in about half of the students. The majority of TTH students reported no increase in headache frequency after analgesic use or increase in the analgesic dose used over time. The main analgesic used was paracetamol (46.5%), followed by ibuprofen, acetaminophen, aspirin, and diclofenac sodium (33.8%, 13.9%, 3%, and 2.8%, respectively).

**Table 3:** Distribution of participants with TTH according to tension headache characteristics, Taif University, KSA, 2017

Variable	Students with TTH (*n* = 396)	
	No.	%
Headache affecting daily activities		
- Yes	18	4.5
- No	378	95.5
Family history of TTH		
- Yes	172	43.4
- No	224	56.6
Frequency of TTH per month		
- Daily	12	3.0
- Fewer than daily to weekly	30	7.6
- Fewer than weekly to monthly	284	71.7
- Fewer than monthly to 1 year	70	17.7
Seeking medical care for TTH		
- Yes	49	12.4
- No	347	87.6
Advice to taker analgesic was by		
- Physician	34	8.6
- Pharmacist	30	7.6
- Family members	275	69.4
- Others: friends, colleagues, neighbors	57	14.4
Frequency of analgesic use		
- Daily	12	3.0
- Fewer than daily to weekly	154	38.9
- Fewer than weekly to monthly	192	48.5
- Fewer than monthly to 1 year	38	9.6
Increase in headache frequency after analgesic use		
- Yes	96	24.2
- No	300	75.8
Increase analgesic dose used over time		
- Yes	41	10.4
- No	355	89.6
Type of analgesic used		
- Paracetamol	184	46.5
- Ibuprofen	134	33.8
- Acetaminophen	55	13.9
- Aspirin	12	3.0
- Diclofenac sodium	11	2.8

**Fig. 1:**
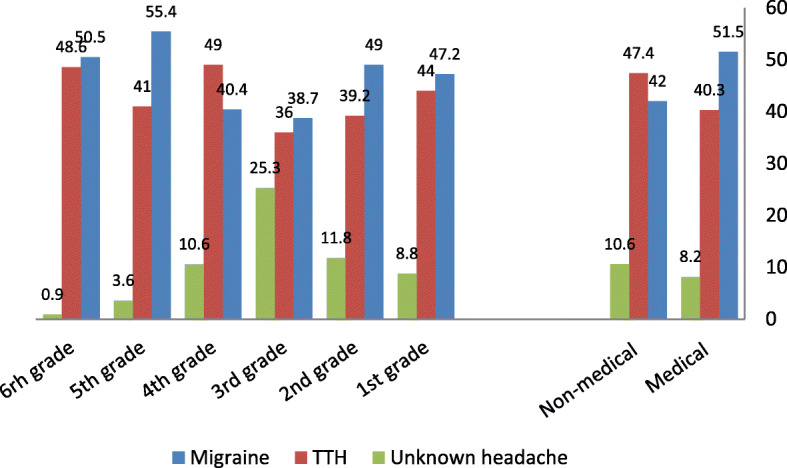
Relationship between college students’ grade and types of headache. N.B. For college type: (χ2 = 8.071 & *p*-value = 0.018). N.B. for students’ grades (*χ*^2^ = 76.59 and *p* value < 0.001)

Figure 1 shows that a significant difference was found between medical and non-medical students according to the prevalence of different types of headache, with medical students showing significantly higher levels of all types of headache (*χ*^2^ = 8.071 and *p* = 0.018). According to the students’ grades, the first and older grades showed a significantly higher prevalence of both migraine and TTH (*χ*^2^ = 76.59 and *p* ≤ 0.001).

**Fig. 2:**
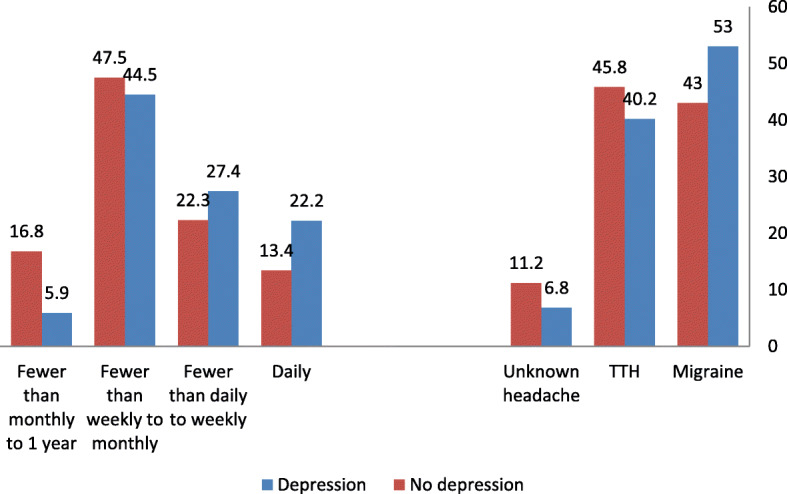
Relationship between depression, headache types, and headache frequency. N.B. for headache types (*χ*^2^ = 11.1 and *p* value = 0.003). N.B. for headache frequency (*χ*^2^ = 26.6 and *p* value < 0.001)

Figure 2 shows that the depression prevalence was significantly higher among migraineur students compared to students with TTH and unknown headache (51.8% vs 43.4% and 34.5%) (*χ*^2^ = 11.1 and *p* = 0.003). Depression prevalence was also significantly higher among students who suffered higher headache frequencies (*χ*^2^ = 26.6 and *p* ≤ 0.001).

## Discussion

In the present study, 68.4% of students had a headache two to three times in the last 3 months. This result is somewhat in agreement with the results revealed from a study done on university students of both sexes in India (63.9%) [4] and Ethiopia (67.22%) [5]. A nearby prevalence was also revealed from a study done on female students of King Faisal University in KSA, where the prevalence of headache was 77% [7].

Lower headache prevalence was reported from other studies done on university students in Iran (58.7%) [26] and South Africa (50.2%) [27]. These lower rates could be attributed to the diagnosis of headache by a questionnaire followed by a physical examination, while in the present study, diagnosis was based only on the self-administered questionnaire without physical examination.

In the same time, higher headache prevalence was reported from other studies done on university students in Brazil (74.5%) [6] and in Iran (90.5% in males and 88.8% in females) [8]. According to studies done on university students in KSA, higher headache prevalence (92%) was reported from a study done on female university students at Taibah University (2015) [19].

This high prevalence observed in the previous studies could be explained by conducting these studies only on medical students. According to Deleu et al., medical students commonly suffer from emotional stress, poor sleeping, and eating behaviors than the general population because of their academic life, which are common triggers for headache [28].

The prevalence of TTH reported in the present study (29.5%) lies within the range reported from population-based studies which ranged from 12 to 78% [29]. Higher prevalence was reported from a previous Saudi study done on female university students from different colleges where 41.6% of students had TTH [7]. A higher prevalence (58%) was also reported in another Saudi study done at Taibah University, which could be explained by conducting that study on medical students [18].

For migraine prevalence, a higher prevalence was reported in previous Saudi studies done on university students of King Faisal University (58.4%) [7] and Taibah University (61.8%) [19]. The lower prevalence found in our study (32.5%) could be explained by the fact that we only describe a 3-month prevalence. In the same time, lower migraine prevalence was reported from other studies done on university students in other Arab and foreign countries [10, 21], and in a study done in KSA [17].

The variation between our results and others in the prevalence of both migraine and TTH could be explained by the difference in the population studied as our participants were only females. The difference in the methodology used and the sampling criteria and the diagnostic parameters used could also be considered for this variability [30]. In addition, genetic characteristic; environmental, cultural, racial, and climate aspects; triggering factors; and different socioeconomic or nutritional status could explain this variation across countries [31].

In the present study, the main trigger for migraine attacks was stress or anxiety, and 18.3% of migraineurs reported the presence of more than one trigger. This result is going with those revealed from other national and international studies [9, 24, 32].

In the present work, 86.6% of migraineur students and 61.1% of students with TTH reported having a family history, a finding consistently found in previous studies and may highlight the role of genetics in this concern [5, 33, 34].

Seeking medical consultation for headache management was poor; only 11.9% of migraineur students and 12.4% of students with TTH reported seeking medical care. This very low figure was reported in previous studies done in Jordan [33] and Yemen [34]. Low level of headache consultation was also reported in other international studies, which showed that a small percentage of younger generation visit health professionals for their headache [35].

In the present study, the majority of migraineurs and students with TTH took analgesic according to an advice from a family member, and about one fifth of them took analgesic according to an advice from a physician or a pharmacist. Similar results were reported in previous studies done in Arab countries [33].

For the majority of both migraineurs and TTH students, paracetamol was the commonly used analgesic followed by ibuprofen and acetaminophen, a finding which is in agreement with those revealed from various studies [4–6]. The popularity of paracetamol and acetaminophen was explained in the previous studies by their low price, safety, and less GIT side effects, in addition to their availability as an over-the-counter medication [28, 34].

Medical students in the present study showed significantly higher levels of migraine headache. This result is in line with those revealed from previous national and international studies done on medical students [4, 8, 9, 19]. This significant difference was explained by emotional stress, anxiety, poor lifestyle, and other risk factors that trigger headache among medical students. Of these, the risk factors are the high level of stress they face due to exams, high-level performance, and many years of learning and training [11].

The prevalence varies also by grade level, where the first and older grades (fifth and sixth) showed a significantly higher prevalence of both migraine and TTH. This result could be explained in the light of having 53.6% of the participants from medical colleges. The present result is in agreement with a Kuwaiti study, where the last two grades had a higher prevalence of migraine headache which was explained by the increasing frequency of exams in the last academic years with a subsequent increase of stress and reading hours and can lead to irregular sleeping pattern [11].

A result was observed in a national study done on medical students of King Saud Bin Abdulaziz University, where the last grades showed a higher prevalence of migraine headache. The study explained this result in the light of exposure of students to emotional stress as they start their internship in different hospitals and cities which is a hard and critical time for students as they have to integrate both internship working and studying for the Saudi Medical License Exam (SMLE) [25]. The high prevalence of headache among the first grade was revealed from other studies which was explained by the emotional stresses faced by those students in their first academic year [11].

In the present study, the prevalence of depression among the studied sample was 38.7%. This result is lower than that revealed from previous Saudi studies done on university students, a matter that could be explained by conducting those studies on exclusively medical students who face lots of stressors [19, 35]. On the other hand, lower prevalence of depression was reported in another Saudi study done at King Faisal University, where the prevalence was 24.4% [36]. This could be attributed to conducting the present work on female students only, where females were found to have a higher prevalence of psychiatric disorders due to the hormonal and serotonin effects [37]. In addition, the Saudi Community imposes more restrictions on girls’ more than boys’ behaviors and has lower expectations for girls’ than for boys’ competencies and achievements [38].

Depression prevalence was significantly higher among migraineur students. This result is in line with that observed in other studies which showed that 62.5% of migraineurs had at least one psychiatric comorbidity [14, 16]. Depression prevalence was significantly higher among students who suffered higher headache frequencies, a result that was observed in previous studies, where higher scores of depressive symptoms were associated with recurrent headache attacks [13, 39].

### Limitations

One of the limitations of this study was using self-reported questionnaires for collecting data which were prone to recall bias. Another limitation was being a cross-sectional study which showed the relation between variables without concluding a cause-effect relationship. Longitudinal studies should be encouraged to determine the causality among variables. In addition, medical students contributed to a relatively large number of the sample. This was the result of the higher response rate from medical colleges as their counterparts from non-medical colleges were engaged in the midterm exam at the time of data collection.

## Conclusion and recommendations

This study demonstrated a high prevalence of headache among studied students and an association between headache and depression. The study calls for increasing awareness towards headache and the importance of seeking medical consultation. Management strategies should be planned, and interventions should be implemented to deal with the problem of headache and depression comorbidity.
